# Treating Soldier-Athletes: A Narrative Review of Psychiatric Disorder Management While Optimizing Physical and Mental Performance in US Army Soldiers

**DOI:** 10.7759/cureus.106763

**Published:** 2026-04-10

**Authors:** James C Mooney, Cyrus Bhadha

**Affiliations:** 1 Psychiatry, Dwight D. Eisenhower Army Medical Center, Augusta, USA; 2 Psychiatry, Irwin Army Community Hospital, Fort Riley, USA

**Keywords:** military, performance, prevention, psychiatry, psychology, readiness

## Abstract

The United States (US) Military has a unique population with distinctive demands, including physical and cognitive requirements that require them to function as what the authors term the "soldier-athletes." This article consists of a review and subsequent commentary regarding psychiatric management of service members through this proposed perspective of the "soldier-athletes." The authors utilize their combined experience as army psychiatrists in a narrative review of this population's risk and propose treatment strategies that focus on prevention, holistic assessment, and intentional clinical decisions within evidence-based practices and guidelines that take into account unique variables of this population and prioritize cognitive and physical well-being, of course, after the overarching goals of stabilization and safety. While the focus is on the "soldier-athletes," the authors propose that treatment strategies reviewed and discussed are logically expanded to the population at large, given the strong bidirectional relationship between mental health and physical and cognitive wellness. The authors first focus on a review of treatment considerations within different disorder categories, followed by individual interventions that warrant specific discussion. This article reflects the views of the authors only and not those of the US Army, US government, or any other organization.

## Introduction and background

With a constant focus on readiness and lethality, the United States (US) Military comprised of nearly 2.86 million members [1], of which the US Army is the largest component [2], has many unique requirements when it comes to soldier function. These are not always seen in the general population, especially when it comes to physical and cognitive function. This stems from the Army’s core mission “to fight and win our Nation’s Wars” [3]. A physically fit and ready force is required for this goal, and in this lies a notable parallel with service members and elite athletes; indeed, the military sees each of its members as an athlete. Many interventions in the field of psychiatry for treating psychiatric conditions are not conducive to optimization of physical and cognitive function, such as weight gain, decreased cognitive function, and medication compliance that is difficult to maintain in austere environments. It is in this setting that soldier-athletes require more nuanced treatment that again aligns with the elite athlete population. Tasked with this treatment, the Defense Health Agency’s motto “Pro Cura Militis” (the care of the warrior) and mission to “support our Nation by improving health and readiness” [4] emphasizes this nuanced mission with standards of service outlined in Army Regulation 40-501 (the regulation that defines the medical standards for Army service) [5]. The authors draw on their experience as active-duty psychiatrists in this narrative review and aim to discuss this unique population and specific treatment considerations, especially when it comes to nontherapeutic interventions for the treatment of psychiatric illness. Considerations in treatment of the “soldier-athlete,” a term utilized to orient focus toward the duty of a Soldier to function with athleticism beyond that required of the general population, largely align with broader societies for performance mental health treatment, such as the International Society for Sports Psychiatry and American Board of Sports and Performance Psychiatry [6,7]. The authors aim to guide mental health providers and primary care providers or other specialists in the psychiatric treatment of not just the soldier-athlete but the population at large, given deteriorating population physical health [8], knowing the impact that physical health has on mental well-being.

## Review

Depression

Depressive disorders are common and debilitating in soldiers as they are in the general population. When treating depression in this population, it is important to consider the iatrogenic effects of potential treatments, including medication side effects that can impact performance. It is also important to thoroughly screen for medical etiologies of depression, as well as behaviors that may cause similar neuroendocrinological symptoms, such as overtraining and inadequate nutrition.

For mild or moderate depression, psychotherapy is the initial recommended treatment, and this may be especially relevant in soldiers, where psychotropic medications can potentially cause adverse effects in cognition, metabolism, and physical performance. If depression is severe, causes significant functional impairment, includes suicidal ideation, or is not improving with psychotherapy, then medications are indicated. While the authors do recommend therapy as a first-line treatment, they reinforce that many medications have proven to have minimal negative impact on performance and are an integral part of treatment.

Serotonin reuptake inhibitors (SSRIs) and bupropion are considered first-line options (among others), due to their comparable efficacy to other antidepressant classes with minimal side-effect burden. Escitalopram, sertraline, citalopram, and fluoxetine are preferred agents as they are usually well-tolerated with lower rates of weight gain or sedation compared to paroxetine and tricyclic antidepressants. Serotonin norepinephrine reuptake inhibitors (SNRIs) may be favorable agents for soldiers with depression and chronic pain syndromes, but they are associated with a higher general incidence of side effects than SSRIs. Mirtazapine may be a less favorable option in soldiers as it is associated with weight gain, decreased metabolism, and dyslipidemia [9]. It could, however, have benefits in certain soldier-athletes who may struggle with being underweight or severe insomnia. Bupropion may be preferred in depression with anergia and atypical features, such as hypersomnia and increased appetite. It may be less favorable for depression with prominent anxiety. Weight gain is a commonly reported side effect for antidepressants (with bupropion being a notable exception) and tends to be more associated with chronic use. Amitriptyline, mirtazapine, and paroxetine have the highest association with weight gain, while bupropion and fluoxetine have the lowest association, with bupropion associated with weight loss and fluoxetine weight neutrality [9].

For soldiers who do not respond to first-line pharmacologic agents, the medical literature describes several treatment strategies, including switching agents, combining antidepressants, augmentation with different medications, or procedural treatments, such as electroconvulsive therapy and transcranial magnetic stimulation (TMS). There are also several supplements with evidence for treating depression, including omega-3 fatty acids, l-methylfolate, s-adenosyl methionine, and creatine. Bright light therapy has been shown to be effective in treating depression, especially seasonal depression, both as monotherapy and as an augmenting agent. For soldiers who do not respond to therapeutic doses of multiple antidepressants, we recommend checking antidepressant plasma concentrations or Cytochrome P450 (CYP) genetic testing to assess for variations in metabolism rates. While augmentation with second-generation antipsychotics should not be outright avoided if clinically necessary, given their risk of metabolic syndrome and impact on deployability, we recommend consideration of other, less physically impactful strategies first.

Bipolar disorder

While the soldier-athlete with bipolar disorder (along with psychotic spectrum disorders) would be disqualified from military service per AR 40-501, individuals are often first diagnosed with bipolar disorder in their twenties to early thirties; thus, initial treatment often occurs while an affected individual is in the early stages of active service. If they are able to be stabilized on treatments that minimize impact on health and fitness, their overall prognosis and quality of life can be greatly improved, even if they cannot remain in service. Therefore, when treating soldiers with bipolar disorder, it is especially crucial to consider the impacts of medications on both cognitive and physical performance. Unfortunately, most established treatments for bipolar disorder fall under two classes, mood stabilizers/neuroleptics and antipsychotics (acting as mood stabilizers), with both of these classes associated with weight gain, insulin resistance, altered lipid metabolism, and cognitive side effects [10].

While lithium is often considered the gold standard for treating bipolar mania and in maintenance treatment, its use is often challenging in athletes as it has a narrow therapeutic index and requires tightly regulated blood levels. Lithium levels are greatly influenced by the body’s fluid and electrolyte balance and may reach toxic levels in those with rigorous exercise regimens. Valproic acid, which is another common treatment, especially in acute mania, holds its own risks of weight gain, organ damage, and is contraindicated in pregnancy. 

Among the FDA-approved treatments for bipolar disorder, the antipsychotics aripiprazole, cariprazine, lurasidone, and ziprasidone, and the mood stabilizer lamotrigine tend to have the lowest risk for metabolic syndrome [11,12]. Aripiprazole may be a preferred agent as it has demonstrated efficacy in treating acute mania, mixed states, and maintenance treatment, with less sedation and cognitive side effects compared to other agents [13]. This, of course, comes with the caveat that not all individuals respond to aripiprazole. Lamotrigine is relatively well-tolerated with evidence of efficacy for bipolar depression and maintenance treatment. Carbamazepine is another option in treating acute manic episodes, but may be less effective in preventing relapse of mood episodes [14]. Additionally, given its unique properties as an autoinducer and multiple drug interactions, the authors see limited use for sustainable bipolar disorder treatment. Ziprasidone and lurasidone are useful alternative agents but tend to cause more sedation than the above medications. While it is important to consider the metabolic and cognitive effects of pharmacologic treatments in individuals with bipolar disorders, the utmost priority should be ensuring stabilization, as undertreatment greatly increases the risk of further relapses, functional impairment, and increased morbidity/mortality. If antipsychotics with higher metabolic risks are used, one could consider adjunctive treatment with metformin for the prevention of weight gain and other metabolic side effects. Of note, the same principles can be applied to psychotic disorders.

Posttraumatic stress disorder (PTSD) and anxiety disorders

PTSD is a common condition in the soldier-athlete, given the correlation between military service and combat deployments, where trauma is more likely to be experienced. This would be an oversimplification of trauma in the military, though sexual trauma and other traumatic experiences are also common in soldiers. One of the core physiologic features of PTSD is an amplified sympathetic tone, mediated multifactorially but in large part due to overactivity of the amygdala via noradrenergic mechanisms. The most efficacious treatment for PTSD is an evidence-based trauma-focused therapy, but medications are also a core treatment modality. First-line medications for PTSD are SSRIs and SNRIs [15], though authors suggest that paroxetine, due to its potent antihistaminergic activity, should be deferred for other SSRIs. One strategy the authors have utilized as an augmentation utilizing the theory of sympathetic overdrive is clonidine as an adjunct to those first-line treatments. Clonidine, which dampens sympathetic tone, has been utilized historically as off-label use for nightmares similar to prazosin, but there is emerging evidence for positive benefits in regard to PTSD symptoms more broadly. It also has potential benefits for attention and insomnia. One study not only suggests the aforementioned symptom improvement but also a dose-related response that can guide its use in augmentation [16]. Often, PTSD impacts hypertension, which clonidine can also improve. Clonidine is also known to aid in focus and sleep, which are important variables in high cognitive and physical performance.

SSRIs/SNRIs remain first-line treatments for anxiety disorders, but authors would recommend consideration in mild cases of anxiety disorders, buspirone as well, given its limited side-effect profile and FDA approval for this indication [17]. In regard to as-needed treatment for anxiety, hydroxyzine is commonly utilized for breakthrough anxiety symptoms. While there is data to support its efficacy against a placebo, it carries a risk for elevated rates of sleepiness and drowsiness. Hydroxyzine carries known sedative impacts on function, especially cognitively [18]. Hydroxyzine also, though less than other antihistaminergic medications, does have anticholinergic effects. Anticholinergic burden is associated with poorer cognitive performance, with evidence in those with schizophrenia [19] and the elderly [20]. Hydroxyzine itself is associated with impaired focus and memory [21], especially the next day, and thus, the authors recommend limited use and only for sleep. Frequent use of as-needed medications generally may suggest that optimization of SSRI and therapy intensity/frequency may need to be further optimized. Authors also suggest consideration of propranolol as a potential alternative for performance anxiety, given its strong evidence in that setting [22], though monitoring for bradycardia, especially if utilized around exertion events. It has been discussed as potentially aiding performance as well [23] and is banned in certain professional sports [24]. Authors suggest its use only for indicated symptoms and not for performance enhancement. Though clinically indicated for severe refractory symptoms in generalized anxiety disorder and panic disorder (contraindicated in PTSD), we utilize benzodiazepines as a last resort given their negative impact on physical and cognitive function, risk for addiction/reliance, and negative impact on deployability/readiness. 

Eating disorders

While the authors don’t suggest specific treatment considerations in eating disorders, it is important to recognize that eating disorders are more prevalent among service members than the general population [25], likely related to height and weight standards. Therefore, we emphasize thorough screening, as many instances of disordered clinical and subclinical examples of disordered eating are missed. This should include screening of disordered eating habits, general diet inquiry, exercise regimen, relative energy deficiency in sport (RED-S), and supplement/medication abuse for the sake of weight loss or gain, such as in anabolic steroids. The SCOFF questionnaire is one of the most studied screeners for eating disorders. Multidisciplinary engagement of a soldier, their support systems, primary care, nutrition, and specialists as appropriate, is important in aiding them in their recovery. 

Substance use disorders

While substance use is generally decreased overall in elite athletes relative to the general population [26], it is important to note that this is not necessarily true in the soldier-athlete. Alcohol is the most prevalent substance of abuse (outside of nicotine), and while heavy use is less common among active-duty service members relative to the general population (5.4% compared to 6.7%), binge drinking is higher than in the general population. Several factors contribute to this, including culture and demographic makeup (skewing male and young adult). After separation from the military, alcohol use increases beyond the general population for heavy use [27]. While the authors do not suggest unique treatment modalities, they do emphasize the importance of these trends as well as recommend screening (the authors utilize the Alcohol Use Disorder Identification-Concise (AUDIT-C) and focused clinical interview), education, and preventative measures. Of note, relapse prevention medications have substantial benefit in alcohol use disorder and should be a part of the treatment, especially given the negative impact of addiction on physical and cognitive function and thus readiness. In fact, service members would not be eligible without a waiver for deployment until completion of one year of Substance Use Disorder Clinical Care (SUDCC) programming, for which referrals should be made if positive screening for a substance use disorder. 

Foundational interventions: exercise, healthy diet, supplementation, and sleep optimization

Exercise

Treatment of mental illness in a way that optimizes physical and cognitive function should always begin with preventative measures. Exercise is one of the most researched and established habits, integral to both physical and mental well-being. A prime example of the importance of physical activity for mental health, a meta-analysis of 218 studies found moderate reductions in depression for walking/jogging, yoga, strength training, mixed aerobic exercises, and tai chi [28]. Studies consistently show that exercise is moderately more effective than control interventions for treating symptoms of depression, though it does not tend to separate from psychotherapy or medications regarding efficacy [29]. There is, however, some data suggesting that in mild depression, exercise relative to selective SSRIs resulted in greater sustained benefit, reduced stress, increased vascularity, increased neuronal growth factors, and increased grey matter volume [30]. There is also evidence showing that exercise can be an effective addition to PTSD treatment with a dose-dependent response [31]. A systematic review of exercise in anxiety disorders showed that both aerobic and anaerobic exercise appear effective in reducing anxiety symptoms, although not as strongly as pharmacologic treatments [32].

There are several proposed mechanisms by which exercise improves mood and cognitive function. Psychologically, exercise may promote self-esteem, self-perception, and self-efficacy and enhance social support. Physiologically, exercise influences the release of dopamine, serotonin, and beta-endorphin, neurotransmitters that improve mood, energy, and pain. Exercise enhances neurogenesis and neuroplasticity via the promotion of growth factors such as brain-derived neurotrophic factor (BDNF), vascular endothelial growth factor (VEGF), and insulin-derived growth factor-1 (IGF-1). Exercise limits neuroinflammation by increasing the production of the anti-inflammatory cytokine IL-10, reducing the production of proinflammatory cytokines, such as C-reactive protein (CRP) and interleukin-6 (IL-6). Additional potential benefits may include reduced oxidative stress, hypothalamic pituitary adrenal axis regulation, and several others [33,34].

Given the clear evidence of the importance of physical fitness, not just for optimal performance but for optimal mental functioning and well-being, it is the recommendation of the authors that all patients maintain an individual physical exercise regimen. Strategies for regimens should be individualized to the patient, but education around the benefits of exercise and discussion of individual goals and plans to achieve them should be a part of the care of active-duty service member psychiatric patients. This is especially true for the soldier-athlete in optimizing physical and mental wellness. 

Healthy Diet

Optimizing diet has long had an integral role in improving physical and mental performance. Beyond impact on physical health and fitness, there is ever-growing data on how diet and the gut microbiome strongly impact mental health. This information is vital to service members, and education on ways to optimize diet should also be a common practice in treating mental illness.

Deficits of several nutrients, including vitamin B12 [35], zinc [36,37], selenium [38], iron [39], vitamin D [40], folate [41], magnesium, potassium, vitamin A, vitamin B6, and vitamin C, have long been linked to mood disorders. Leafy greens, cruciferous vegetables, oysters/muscles/seafoods, and animal meats tend to be rich in many of these substances [42]. Omega-3 fatty acids appear to play a notable role in the physiology of brain health, given their evidence in reducing depressive symptoms [43], bipolar symptoms, especially bipolar depression and maintenance treatment [44], and other mental conditions such as attention-deficit/hyperactivity disorder (ADHD), schizophrenia, PTSD, and mood regulation in general, such as in borderline personality disorder [45,46]. Though omega-3 fatty acids have a large body of evidence for aiding treatment of these conditions, they are often insufficient alone, and their role should be more of an adjunctive treatment to established first-line treatments such as psychotherapy and psychopharmacology. The Mediterranean diet, which emphasizes fresh fruits and vegetables, whole grains, legumes, fish, and unsaturated fats, has good evidence for improving cognition and reducing the risk of depression.

There are many plausible mechanisms for how diet affects depression, including anti-inflammatory effects, hormonal modulation, and the production of amino acids. Monoamine neurotransmitters, such as dopamine, serotonin, and norepinephrine, require the amino acids tyrosine and tryptophan, as well as cofactors, including vitamins B6 and C. Omega-3 fatty acids are an integral component of neuronal cell membranes. Avoidance of ultraprocessed foods is also crucial, as they are significantly associated with depression [47]. We advise well-rounded, nutrient-rich, unprocessed foods as a general recommendation to optimize both physical fitness and mental fitness. Research is ever-evolving with diet and mental health, and its impact cannot be overstated. We recommend diet counseling through psychoeducation. Further evidence-based recommendations can be found in patient-friendly literature. Guiding soldier-athletes to resources with evidence-based diet strategies for addressing the micronutrients needed to optimize cognitive function and therefore reduce mental illness is important, and the authors recommend that providers familiarize themselves with appropriate resources for patients [48].

Supplementation

The active-duty population, similar to the general athlete population, is known for supplementation in order to optimize their physical performance. Knowledge of risks and benefits is integral to support healthy and evidence-based use of supplements so soldier-athletes can make informed and health-promoting decisions about supplementation. As mentioned in the diet above, for those for whom omega-3 fatty acid supplementation makes sense, a discussion of risks and potential benefits can be considered. A review of and discussion of any herbal or over-the-counter supplements should be undertaken with patients.

One commonly used supplement, creatine monohydrate, or “creatine”, is an organic compound that plays a crucial role in energy production by helping to regenerate adenosine triphosphate (ATP). It is primarily found in skeletal muscle and brain tissue. It has been widely utilized as a dietary supplement to improve athletic performance, muscle growth, and recovery, but there has been increasing evidence supporting its use to aid cognitive performance and reduce mental health symptoms. Creatine supplementation in placebo-controlled trials has shown efficacy in treating depression when combined with cognitive behavioral therapy [49] or when added to SSRIs [50]. In addition to boosting mood, creatine may boost brain energy metabolism and neuroplasticity. Studies have shown that compared to a placebo, creatine supplementation significantly improved cognitive performance, especially short-term memory and reasoning [51,52]. Creatine may also have neuroprotective effects, protecting neurons from inflammation and oxidative damage [53,54]. Most studies of creatine tend to have small sample sizes and focus primarily on unipolar depression, but there is some evidence of benefit in bipolar depression, though with potential risk of hypomanic or manic switch [55,56], in anxiety as a secondary outcome [57], and in PTSD [58]. Creatine is generally well-tolerated, easily accessible, and widely considered safe for long-term use [59,60]. Overall, the existing literature on psychiatric applications of creatine is limited but promising, and the authors recommend continuing to monitor emerging research on this topic. At this time, the authors generally support creatine supplementation, though they would caution against use in those at high risk for bipolar disorders and those with pre-existing renal disease.

Optimizing Sleep

It is well-known that sleep deprivation can reduce cognitive function, worsen mental health conditions, reduce physical performance, and increase the risk of accident/injury. Sleep disruption is highly prevalent throughout society, including soldier-athletes. Soldier-athletes often have to cope with operational demands, irregular work schedules, frequent deployments and relocations, and high-stress environments, which can negatively impact sleep. They may feel pressure to use stimulants, such as caffeine, to balance training with academic or work demands.

When individuals present with sleep issues, we recommend initially screening them for sleep disorders, such as obstructive sleep apnea (OSA), restless legs syndrome, and parasomnias. For patients with insomnia, primary treatments include sleep hygiene optimization, relaxation techniques, and cognitive behavioral therapy for insomnia (CBT-I). Part of performance optimization is the optimization of sleep beyond the minimum recommended hours. Utilizing data from elite athletes, where increasing total sleep time by roughly one hour daily (at night or through naps), even in those who get the low end of recommended hours of sleep (~7 hours), can statistically improve performance [61].

In the soldier-athlete population, it is the author’s recommendation that medications are generally reserved for short-term, as-needed treatment of insomnia that does not improve with behavioral therapies, as they may cause adverse effects such as morning sedation, impaired cognitive function, and have potential for dependence. When medications are considered, we prefer the temporary use of melatonin or ramelteon, a melatonin receptor agonist, as both tend to have fewer adverse effects on cognition and physical performance compared to other medications. Melatonin and its derivatives are also known to improve sleep and regulate circadian rhythms in both shift workers and individuals with jet lag, suggesting potential benefits for those with interrupted sleep due to travel or shift changes. If sedatives with primarily antihistaminergic mechanisms, such as doxepin or hydroxyzine, are considered, we recommend educating patients about the potential for unwanted metabolic or anticholinergic side effects, such as weight gain, dry mouth, and cognitive impairment, as well as counseling them about the risk of tolerance. We recommend against regular use of Z-drugs or benzodiazepines as they may have next-day impacts on cognitive/physical performance, in addition to risks for parasomnias, tolerance, and dependence.

Other treatments of consideration: biofeedback and TMS

Biofeedback

Biofeedback, or the technique of gaining greater awareness of one's own body function using instruments, with a goal of being able to achieve greater control over bodily, emotional, and cognitive function, has been utilized for some time in both behavioral health and performance optimization. Biofeedback has many beneficial indications for the soldier-athlete, both from cognitive and physical aspects. Pain [62], including phantom limb pain [63] and migraines [64], general stress and anxiety [65], and depression [66] have all been shown to improve with biofeedback training. Given not only improvement of these symptoms but direct benefit in performance independent of psychopathologic improvement [67], biofeedback has the potential to be an outstanding tool with few to no downsides in treating the soldier-athlete. In the opinion of the authors, it is a highly underutilized modality that should be incorporated more frequently.

TMS

TMS is a treatment of psychiatric illness where a magnetic field is utilized to stimulate neuronal firing/activation [68]. It is an FDA-approved treatment for major depressive disorder (MDD), as first established in 2008. Since that time, it has been approved in various forms for migraines, obsessive compulsive disorder (OCD), smoking cessation, depressive episodes other than within MDD, and anxiety that is associated with depressive episodes [69]. Off-label usage with varying levels of supportive data exists for the rehabilitation of speech and motor loss following neurological injury and mitigating chronic pain [70]. There exists promising data in PTSD and bipolar depression [71], though further study is important to establish efficacy. In regard to soldier-athletes, TMS is an approved treatment modality for depression by TRICARE and is directly available at multiple military hospitals or military treatment facilities (MTF). It is also available at nearly all duty locations through community referral. The authors suggest TMS as a viable option for soldier-athletes for a few key reasons. For one, the treatment has strong evidence for efficacy relative to placebo [72], with ongoing comparison studies compared to SSRIs [73]. Secondly, TMS’s side-effect profile is not only widely tolerable but has minimal impact on physical/mental function relative to most other treatment modalities, especially other interventional modalities such as esketamine and electroconvulsive therapy (ECT), including even well-tolerated medication management strategies. Primary side effects are mild in severity and include headache or brief local discomfort. More severe side effects are very rare and include seizures, hearing impairment, and initiating manic flip in individuals with bipolar disorder [74]. Given established efficacy in the setting of minimal negative side effects, authors consider this an underutilized treatment for soldier-athletes, often limited by availability and/or coverage (but, for example, TRICARE (the insurance that covers military members, retirees, and their families) requires only two failed medication trials for approval) [75]. Of note, acoustic stimulus may be triggering for those struggling with hyperarousal in PTSD, though advancements in machines have greatly minimized this risk. 

Discussion

In all, a holistic, nuanced, and individualized approach should be undertaken in caring for soldier-athletes to include their physical well-being prominently, given its importance to their mission, readiness, and mental well-being. The authors aim to discuss considerations of treatment with this principle in mind, which are consistent with consensus statements by experts in sports psychiatry and psychology [76-78], given the physical requirements of soldier-athletes that mimic collegiate and professional athletes. Education is key in this mission, not just for soldier-athletes but also for the systems they exist in and their providers. This is verified with a systematic review showing that service members have increased risks for mental disease and increased barriers [79]. While treatment principles discussed focus on active duty soldier-athletes, the general principles within treatment guidelines focusing on holistic health and optimization of physical and cognitive function can be beneficial for all suffering from mental illness.

## Conclusions

The US Military has a unique population and mission that requires its members to be at or as close to optimal physical and cognitive performance. Treating psychiatric conditions is in and of itself important in order to achieve this goal, but doing so in a manner that is holistic and prevention-focused and minimizes any negative impact from the treatments themselves is especially important. Many soldiers receive care from providers outside the military-trained provider network, and this narrative review aims to educate anyone who may treat an active-duty service member and to view them through the lens of the "soldier-athlete." While treatment recommendations are directed at soldier-athletes, the concept of holistic treatment that attempts to optimize cognitive and physical function while treating mental health illness within treatment guidelines can be translatable to the population as a whole. This is especially true given the strong bidirectional relationship that physical and cognitive health has with mental health and mental illness alleviation. 
